# Predictive and Prognostic Role of PD-L1 in Urothelial Carcinoma Patients with Anti-PD-1/PD-L1 Therapy: A Systematic Review and Meta-Analysis

**DOI:** 10.1155/2020/8375348

**Published:** 2020-06-27

**Authors:** Haoran Liu, Tao Ye, Xiaoqi Yang, Peng Lv, Xiaoliang Wu, Hui Zhou, Hongyan Lu, Kun Tang, Zhangqun Ye

**Affiliations:** Department of Urology, Tongji Hospital, Tongji Medical College, Huazhong University of Science and Technology, Wuhan 430030, China

## Abstract

Recently, checkpoint inhibition of the PD-1/PD-L1 axis has been shown to be therapeutically relevant in urothelial carcinoma (UC). To evaluate the predictive and prognostic value of PD-L1 on response and survival in UC patients after cystectomy, chemotherapy, or anti-PD-1/PD-L1 therapy, a systematic review of PubMed, Embase, Web of Science, and the Cochrane Library was performed. A total of 2154 patients from 14 published studies were included. In all UC patients after cystectomy, tumour cell (TC) PD-L1 expression was not associated with the OS or PFS. For the subset of patients with organ-confined disease, TC PD-L1 expression significantly predicted OS after cystectomy (*P* = 0.0004). There was no significant evidence of an association between TC PD-L1 status and ORR or OS for UC patients treated with platinum-based chemotherapy. For UC patients treated with anti-PD-1/PD-L1 therapy, TC PD-L1 expression ≥ 5% could predict the response (*P* = 0.005), but not for the 1% cut-off (*P* ≥ 0.05). As for PD-L1 expression in tumour-inflating immune cells (TIICs), both subsets with IC2/3 vs. IC0/1 and IC1/2/3 vs. IC0 were associated with ORR to anti-PD-1/PD-L1 therapy. In the TIIC subset, IC2/3 vs. IC0/1 of PD-L1 was associated with higher CR (*P* = 0.002), PR (*P* = 0.04), and PD (*P* = 0.007). Further, higher TIIC PD-L1 status benefited from longer PFS (*P* < 0.001), but was not associated with OS in UC patients with anti-PD-1/PD-L1 therapy. Our study suggested that TIIC PD-L1 expression with 5% cut-off was valuable as a predictive and prognostic biomarker for ORR and PFS in UC patients with anti-PD-1/PD-L1 therapy.

## 1. Introduction

Urothelial carcinoma (UC) is regarded as an aggressive tumour, with unfavorable clinical survival in advanced stages and metastatic diseases. Radical cystectomy (RC) is the gold-standard treatment for muscle-invasive organ-confined UC, providing efficacy of local control and better disease-free survival (DFS) [1, 2]. With high expression level of programmed death-ligand 1 (PD-L1), UC appears to be immunogenic, demonstrating the potential value of PD-L1 as a promising biomarker in UC after RC [3]. Although the upregulated PD-L1 was associated with tumour-infiltrating immune cell (TIIC) response and advanced disease, clinical efficiency for UC patient survival was characterized by different degrees of uncertainty [4].

Patients with metastatic UC usually had a poor prognosis; perioperative cisplatin-based chemotherapy in addition to RC could benefit a response of 50% and prolong survival [5]. However, Tsao and colleagues performed a meta-analysis raising serious doubt about the predictive value of PD-L1 expression in prognosis and response for adjuvant chemotherapy in early stage non-small cell lung cancer (NSCLC). They indicated that PD-L1 status showed neither prognostic nor predictive value of benefits from adjuvant chemotherapy in patients with partial pneumonectomy [6]. Therefore, it remained contentious whether PD-L1 could serve as a valuable biomarker in UC patients with adjuvant chemotherapy.

Recently, blocking immune checkpoints with anti-PD-1/PD-L1 monoclonal antibodies has demonstrated promising clinical efficacy for advanced UC [7]. The effect of PD-1 on T-cells with its ligand PD-L1 on tumour cell and immune cell interaction inhibited the function of effector T-cells [8]; therefore, tumours could escape from T-cell regulated immune response by blocking the PD-1/PD-L1 signaling pathway [9]. PD-1/PD-L1 inhibitors have shown survival benefits in various advanced cancers, including melanoma, lymphoma, NSCLC, renal cell carcinoma, and UC [9–12]. PD-L1 status has been demonstrated to significantly correlate with response and survival improvement from anti-PD-1/PD-L1 immunotherapy in UC patients [13], while there is no convincing evidence whether PD-L1 expression in tumour cells (TCs) or TIICs with a cut-off value of 5% or 1% could predict the prognosis and response.

To clarify the available evidence, we conducted this meta-analysis of eligible literatures to determine the predictive and prognostic significance of PD-L1 expression in UC patients receiving cystectomy, chemotherapy, or anti-PD-1/PD-L1 immunotherapy.

## 2. Material and Methods

### 2.1. Search Strategy

**Figure 1:**
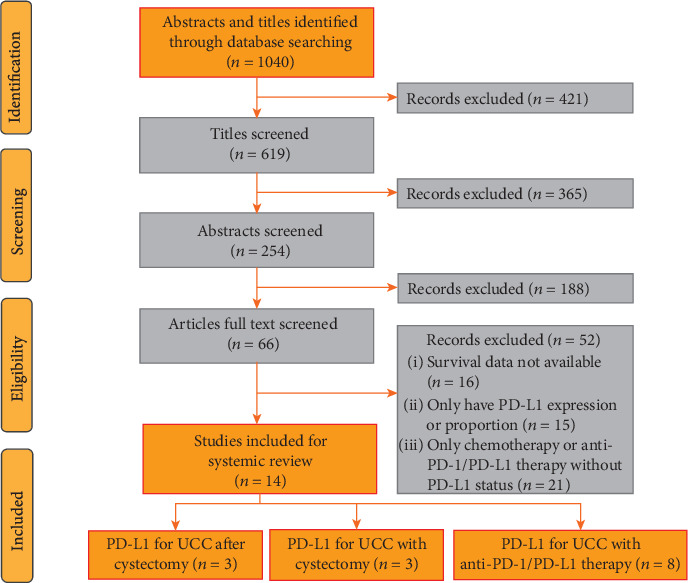
Flow diagram for study selection.

All methods for this systematic review and meta-analysis are outlined in a prospectively registered protocol available online (PROSPERO identifier CRD42019130411). Our meta-analysis was performed following the Preferred Reporting Items for Systematic Reviews and Meta-Analyses (PRISMA) guidelines [14]. Studies published before January 2019 were electronically retrieved from the PubMed, Web of Science, Embase, and Cochrane Library databases. The following terms were used: urothelial carcinoma or urothelial tumour or urothelial neoplasm or bladder cancer or bladder tumour; PD-L1 or programmed cell death ligand 1 or B7-H1 or CD274. The reference lists were also screened to obtain other eligible studies by correspondence with study investigators. Each study was evaluated independently by two reviewers for the inclusion. Any disagreement in the articles was resolved by discussing with a third reviewer.  Figure 1 shows the flow diagram of the study selection.

### 2.2. Selection Criteria

Included publications should satisfy the following criteria: (1) The studies reported PD-L1 expression on urothelial carcinoma. (2) The results showed the association of PD-L1 status and any of the following outcomes: objective response rate, PFS or OS after cystectomy, and chemotherapy or anti-PD-1/PD-L1 immunotherapy. (3) Only clinical trials, including prospective or retrospective cohort studies or comparative series, were eventually enrolled. Exclusion criteria were the following: (1) studies only reported the PD-L1 expression in urothelial carcinoma patients; (2) studies only studied the molecular mechanism of PD-L1 and its biological function in bladder cancer; (3) animal or in vitro studies; (4) studies did not report or no data available on response rate, PFS, or OS; and (5) articles not in English, case reports, comments, letters, editorials, congress reports, and review papers. When several papers from the same center were available, the one with the newest information, the longest follow-up, and the most participants was finally included in our meta-analysis.

### 2.3. Data Extraction and Study Quality

The following data was extracted independently by two trained reviewers (TK and LHR) using a prior-designed form: last name of authors; publication year; study design; country; participants' inclusion and exclusion criteria; mean or median age of participants; sample size; tumour stage; PD-L1 status; assigned to treatment with cystectomy, chemotherapy, or anti-PD-1/PD-L1 immunotherapy; length of follow-up; and primary endpoints including response rate, PFS, and OS. Missing, unclear, but important supplementary data were requested from primary study authors. All discrepancies were adjudicated by a third reviewer and solved by discussion (YZQ). RevMan software version 5.3. (Cochrane, London, UK) was used to perform risk of bias graph and summary.

### 2.4. Statistical Analysis

All data analysis was conducted using RevMan software version 5.3. Efficacy data from assigned patients were calculated on an intention-to-treat basis in all enrolled studies [15]. The concerned endpoints included the response rates, PFS, and OS. In terms of both OS and PFS, the pooled HRs and their 95% CI were calculated. A subgroup analysis was performed for patients receiving anti-PD-1/PD-L1 immunotherapy by PD-L1 cut-off value (IC2/3 vs. IC0/1 or IC1/2/3 vs. IC0). Statistical heterogeneity was quantified by the *Q* and *I*^2^ tests. Based on the absence or presence of interstudy heterogeneity, pooled odds ratio (OR) and hazard ratio (HR) estimates were obtained by use of a fixed or random effects model. *P* values < 0.05 indicated statistical significance.

## 3. Results

### 3.1. Baseline Characteristics

Fourteen publications were selected for inclusion; three trials reported the association of PD-L1 status and concerned endpoints in UC patients after cystectomy, three trials reported platinum-based chemotherapy, and eight trials reported anti-PD-1/PD-L1 immunotherapy (Figure 1). All the included studies detected PD-L1 expression by immunohistochemistry (IHC), eight studies only detected the PD-L1 expression in tumour cells (TCs), four trials only in tumour-inflating immune cells (TIICs), and two studies measured PD-L1 in both locations. Three studies recruited UC patients after cystectomy, and one study was excluded without reporting survival HR value and 95% CI [16]. Two of three studies evaluated PD-L1 expression both in all UC patients and organ-confined disease. Three studies reported OS, and two showed CSS and DFS (Table 1). The baseline characteristics of three studies that reported chemotherapy are outlined in Table 2, and all three studies were about platinum-based preoperative treatment. For patients receiving anti-PD-1/PD-L1 immunotherapy, most of the published trials set 1% or 5% as the cut-off value, and one study was excluded with a cut-off value of 25% [17]. Among the eight trials, only one study was prospective but nonrandomized, and the others were multicenter RCTs. Four studies used atezolizumab, two used nivolumab, one used avelumab, and one used pembrolizumab. PD-L1-positive proportion has been noted ranging from 10.8% to 46.2% (5% cut-off) and 37.3% to 81.5% (1% cut-off) in UC patients. The ORR to anti-PD-1/PD-L1 immunotherapy in UC patients with PD-L1-positive expression ranged from 26.0% to 43.3% (5% cut-off) and 17.9% to 30.2% (1% cut-off) (Tables 3 and 4).

### 3.2. PD-L1 Expression in TCs Predicted Poor Survival after Cystectomy for Patients with Organ-Confined Tumours (but Not All UC)

**Figure 2:**
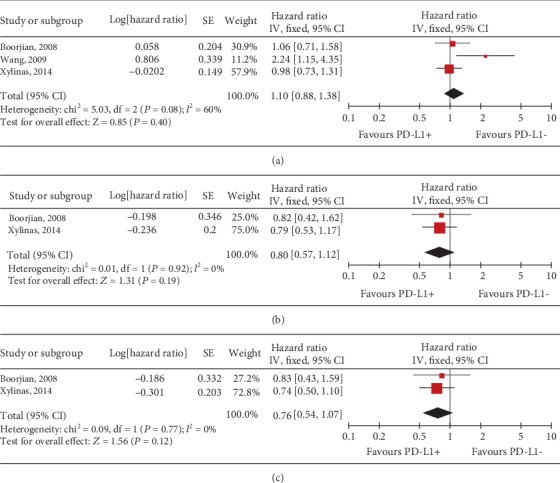
Forest plots of tumour cell PD-L1 expression predicted (a) OS, (b) CSS, and (c) DFS for all UC patients after cystectomy. PD-L1: programmed death-ligand 1; +/-: positive/negative; OS: overall survival; CSS: cancer-specific survival; DFS: disease-free survival; CI: confidence interval.

**Figure 3:**
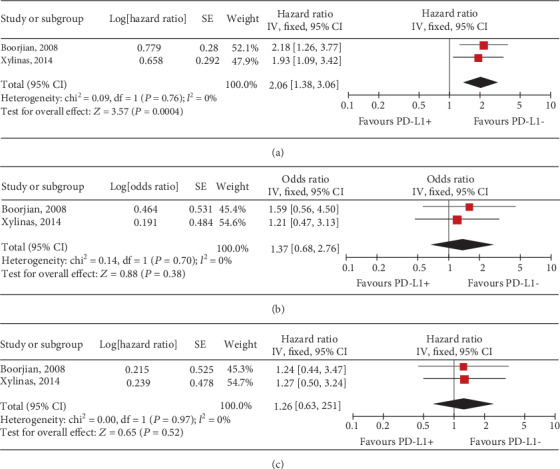
Forest plots of tumour cell PD-L1 expression for predicting (a) OS, (b) CSS, and (c) DFS for patients with organ-confined tumours after cystectomy. PD-L1: programmed death-ligand 1; +/-: positive/negative; OS: overall survival; CSS: cancer-specific survival; DFS: disease-free survival; CI: confidence interval; IV: inverse variance; SE: standard error.

Three studies considering OS and CSS as the primary endpoints were included [4, 18, 19]. For all patients treated with cystectomy, pooled results indicated that the expression of PD-L1 in TCs was not related with the OS (HR, 1.10; 95% CI, 0.88-1.38; *P* = 0.40; Figure 2(a)), CSS (HR, 0.80; 95% CI, 0.57-1.12; *P* = 0.19; Figure 2(b)), or PFS (HR, 0.76; 95% CI, 0.54-1.07; *P* = 0.12; Figure 2(c)). However, for organ-confined UC, PD-L1 expression in TCs significantly predicted all-cause mortality (OS) after cystectomy (HR, 2.06; 95% CI, 1.38-3.06; *P* = 0.0004; Figure 3(a)), but was not significant in terms of CSS (HR, 1.37; 95% CI, 0.68-2.76; *P* = 0.38; Figure 3(b)) and DFS (HR, 0.26; 95% CI, 0.63-2.51; *P* = 0.52; Figure 3(c)).

### 3.3. TC PD-L1 Status Showed No Obvious Relationship with the Prognosis or Response to Platinum-Based Chemotherapy of UC Patients

**Figure 4:**
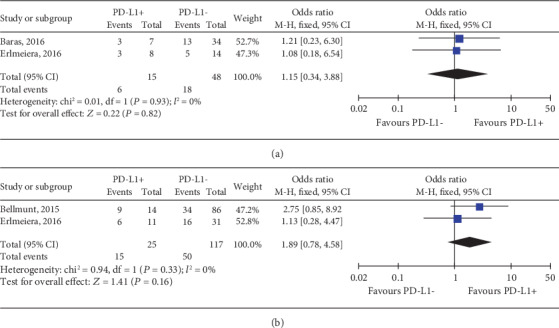
Forest plots of tumour cell PD-L1 status for predicting the (a) response and (b) prognosis to platinum-based chemotherapy in UC patients. PD-L1: programmed death-ligand 1; +/-: positive/negative; ORR: objective response rate; OS: overall survival; CI: confidence interval; M-H: Mantel-Hansel; SD: standard deviation.

In terms of the previously described association of PD-L1 expression and clinical outcomes in UC, it was investigated whether pretreatment PD-L1 status could predict the response to chemotherapy from three eligible studies [3, 20, 21]. As shown in Figure 4(a), there was no statistical difference of PD-L1 status in the tumour cell membrane between responders and resistant cases (OR, 1.15; 95% CI, 0.34-3.88; *P* = 0.82). Also, the result showed no significant evidence of a relationship with PD-L1 expression and OS (OR, 1.89; 95% CI, 0.78-4.58; *P* = 0.16; Figure 4(b)) for UC patients receiving platinum-based chemotherapy.

### 3.4. TC PD-L1 Expression Failed to Predict the Response to Anti-PD-1/PD-L1 Immunotherapy in UC Patients

**Figure 5:**
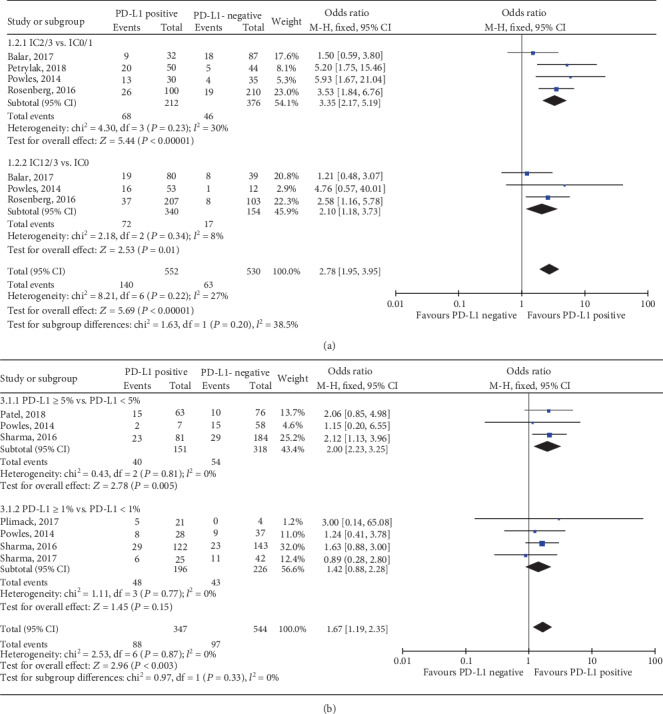
Forest plots of (a) immune cell and (b) tumour cell PD-L1 status with cut-off values of 5% and 1% in predicting the response to anti-PD-1/PD-L1 immunotherapy. PD-L1: programmed death-ligand 1; ORR: objective response rate; IC: immune cell; CI: confidence interval; M-H: Mantel-Hansel; SD: standard deviation.

**Figure 6:**
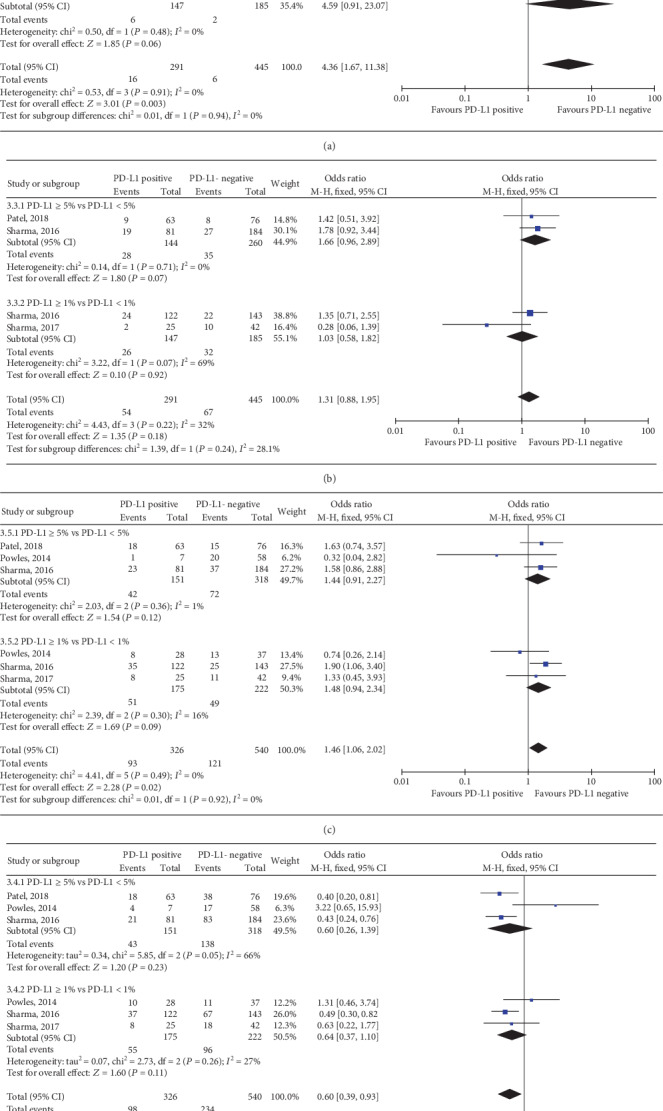
Forest plots of tumour cell PD-L1 status with cut-off values of 5% and 1% in predicting the (a) CR, (b) PR, (c) SD, and (d) PD to anti-PD-1/PD-L1 immunotherapy. PD-L1: programmed death-ligand 1; CR: completed response; PR: partial response; SD: stable disease; PD: progressive disease; CI: confidence interval; M-H: Mantel-Hansel; SD: standard deviation.

Among the 8 trials, 5 RCTs of elevated TC PD-L1 status predicting response to the PD-1/PD-L1 blockade therapy were pooled in this meta-analysis [22–26]. TC PD-L1 status with 5% as the cut-off value could predict the ORR to anti-PD-1/PD-L1 immunotherapy in UC patients (Figure 5(b)). TC PD − L1 ≥ 5% was correlated with higher completed response (CR) (OR, 4.24; 95% CI, 1.29-13.96; *P* = 0.02; Figure 6(a)). Otherwise, TC PD-L1 status with a cut-off value of 5% but not 1% failed to predict the partial response (PR), stable disease (SD), or progressive disease (PD) (Figure 6) in UC patients receiving anti-PD-1/PD-L1 immunotherapy.

### 3.5. TIIC PD-L1 Expression Predicted Response to Anti-PD-1/PD-L1 Immunotherapy in UC Patients

**Figure 7:**
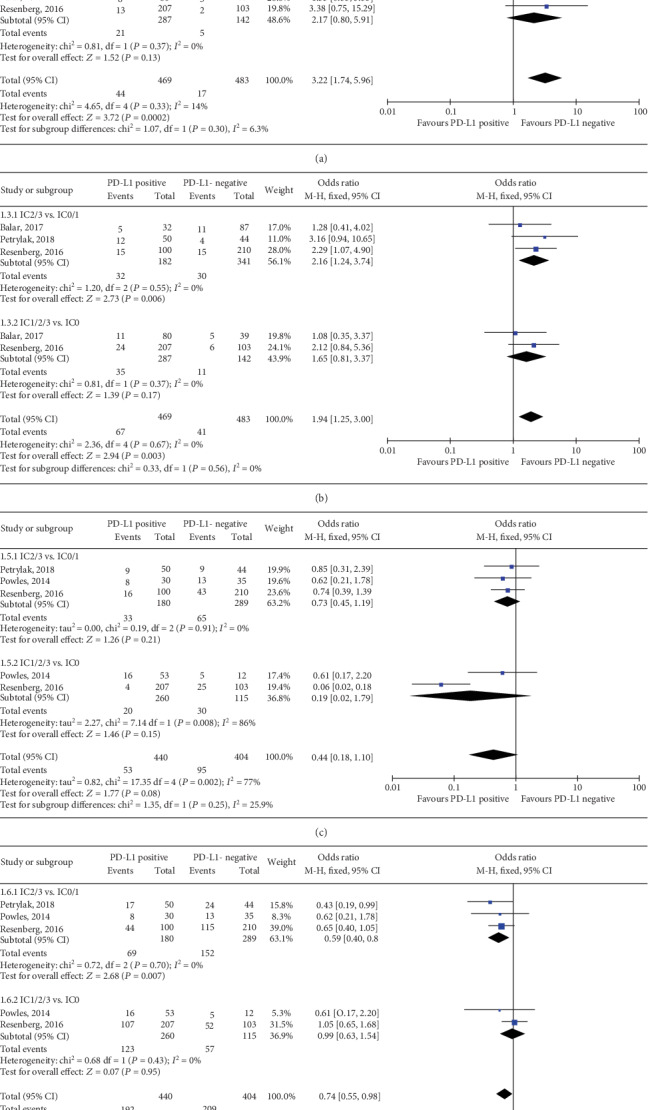
Forest plots of immune cell PD-L1 status with cut-off values of 5% and 1% in predicting the (a) CR, (b) PR, (c) SD, and (d) PD to anti-PD-1/PD-L1 immunotherapy. PD-L1: programmed death-ligand 1; IC: immune cell; CR: completed response; PR: partial response; SD: stable disease; PD: progressive disease; CI: confidence interval; M-H: Mantel-Hansel; SD: standard deviation.

Among the 6 trials, 4 RCTs of elevated TIIC PD-L1 status predicting the response to anti-PD-1/PD-L1 immunotherapy were pooled in this meta-analysis [13, 24, 26, 27]. Both TIIC PD-L1 status with IC2/3 vs. IC0/1 and IC1/2/3 vs. IC0 showed significant relationship with ORR to anti-PD-1/PD-L1 immunotherapy and a higher PD-L1 expression was correlated with a better response (IC2/3 vs. IC0/1: OR, 3.35; 95% CI, 2.17-5.19; *P* < 0.001; Figure 5(a); and IC1/2/3 vs. IC0: OR, 2.10; 95% CI, 1.18-3.73; *P* = 0.01; Figure 5(b)). In the TIIC subset, the positive expression (score of 2-3) versus the negative (score of 0-1) of PD-L1 was correlated with higher CR (OR, 4.21; 95% CI, 1.97-9.02; *P* = 0.0002; Figure 7(a)), PR (OR, 2.16; 95% CI, 1.24-3.74; *P* = 0.006; Figure 7(b)), and PD (OR, 0.59; 95% CI, 0.40-0.87; *P* = 0.007; Figure 7(d)); however, it could not predict the SD (OR, 0.73; 95% CI, 0.45-1.19; *P* = 0.21; Figure 7(c)). In the subset of IC1/2/3 vs. IC0, results indicated that PD-L1 expression level had no significant association with the CR (OR, 2.17; 95% CI, 0.80-5.91; *P* = 0.13; Figure 7(a)), PR (OR, 1.65; 95% CI, 0.81-3.37; *P* = 0.17; Figure 7(b)), SD (OR, 0.19; 95% CI, 0.02-1.79; *P* = 0.15; Figure 7(c)), or PD (OR, 0.99; 95% CI, 0.63-1.54; *P* = 0.95; Figure 7(d)).

### 3.6. TIIC PD-L1 Status Predicted PFS and OS in UC Patients Receiving Anti-PD-1/PD-L1 Immunotherapy

**Figure 8:**
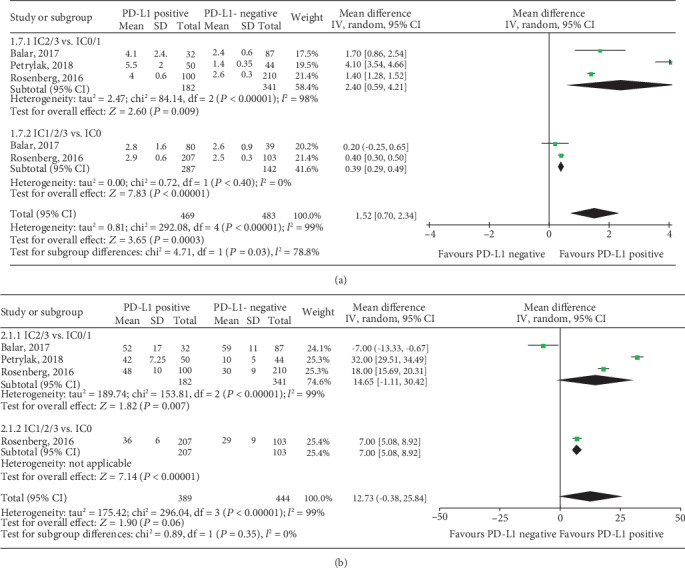
Forest plots of immune cell PD-L1 status with cut-off values of 5% and 1% in predicting the (a) PFS and (b) OS for UC patients with anti-PD-1/PD-L1 immunotherapy. PD-L1: programmed death-ligand 1; IC: immune cell; PFS: progression-free survival; OS: overall survival; CI: confidence interval; IV: inverse variance; SD: standard deviation.

Further, only three trails reported the relationship of TIIC PD-L1 status and prognosis in UC patients receiving anti-PD-1/PD-L1 immunotherapy [13, 27, 28]. As shown in Figure 8, pooled results showed that elevated TIIC PD-L1 expression level benefited from improved PFS (IC2/3 vs. IC0/1: WMD, 2.40; 95% CI, 0.59-4.21; *P* = 0.009; and IC1/2/3 vs. IC0: WMD, 0.39; 95% CI, 0.29-0.49; *P* < 0.001), but was not correlated with OS in UC patients.

## 4. Discussion

Currently, this was the first meta-analysis discussing the predictive and prognostic significance of PD-L1 expression in UC patients treated with cystectomy, chemotherapy, or anti-PD-1/PD-L1 immunotherapy. We confirmed that TC PD-L1 status could predict reduced survival after cystectomy for organ-confined UC patients, but not all UC patients. However, we found that TC PD-L1 expression was neither a predictive biomarker for survival benefit or response to adjuvant platinum-based chemotherapy nor a biomarker for response to anti-PD-1/PD-L1 immunotherapy. At the same time, we demonstrated that higher PD-L1 expression of TIICs but not TCs showed significant relationship with better response to the PD-1/PD-L1 blockade therapy. Furthermore, our results found that immune cell PD-L1 expression could serve as a prognostic biomarker for PFS but not OS in patients receiving anti-PD-1/PD-L1 immunotherapy.

Evidence suggested that UC was an immunogenic disease; in addition, presence of tumour-infiltrating lymphocytes (TILs) often correlated with immune response against the tumour and favorable clinical outcomes [29]. Aberrant expression of T-cell coregulatory molecule PD-L1 interacted with T-cell PD-1 that resulted in tumour-specific T-cell apoptosis, which might evade host immune surveillance, and was related with unfavorable outcomes in tumours [30]. Furthermore, PD-L1-positive expression has demonstrated a significant correlation with increased risk of disease progression and cancer death in various tumours [31–33]. Previously, two meta-analyses focused on PD-L1 and UC survival were reported. Wu et al. [34] indicated that PD-L1 status was related with worse 3-year overall survival in UC, and Wang et al. [35] revealed that PD-L1 status could predict the clinical stage of UC. Our pooled results with HR value and 95% CI raised a doubt and showed different results with them. We demonstrated that TC PD-L1 status was not correlated with the OS, CSS, or DFS in UC patients treated with cystectomy. However, for organ-confined UC, TC PD-L1 status could predict OS after cystectomy. Our findings were consistent with the published results of Boorjian et al. [4] and Xylinas et al. Increased tumour cell PD-L1 expression was related with advanced tumour stage, which could be an explanation for the predictive role of mortality in organ-confined but not local control tumours [4]. Of note, no study elevated the prognostic significance of immune cell PD-L1 status for UC patients receiving cystectomy, especially for early-stage tumours. Therefore, further trials are needed to explore whether PD-L1 expression in the immune cell has a prognostic role for UC patients who underwent cystectomy.

The recommended first-line therapy for metastatic UC is cisplatin-based chemotherapy, and nearly 50% of patients could respond to the treatment [36]. The improved prediction of clinical outcomes for advanced UC patients with platinum-based chemotherapy has recently attracted great interest [37, 38]. A recent RCT failed to demonstrate the p53 mutation either as a predictive biomarker of survival or as a response to adjuvant chemotherapy in UC patients who underwent RC [39]. PD-L1 status could predict postoperative outcomes in organ-confined UC patients and might provide better implications for the management of metastatic UC patients with chemotherapy. Our pooled results found that the expression of PD-L1 had no association with prognostic or predictive benefit from platinum-based chemotherapy. It was similar with that of Tsao et al. [6], who performed a pooled analysis using three pivotal adjuvant chemotherapy trials, and found that TC PD-L1 had neither prognostic nor predictive value from adjuvant chemotherapy in NSCLC. We concluded the nonsignificant role of tumour cell PD-L1; however, further trials are needed to assess whether PD-L1 expression in the immune cell could be a prognosticator for UC patients with chemotherapy.

Cisplatin-based chemotherapies were associated with several substantial toxicities, and only 10% of participants responded to the second-line single-agent chemotherapy [6]. Immune checkpoint blockade is a promising new way to cancer therapy via the activation of therapeutic tumour immunity. It was reported that PD-1/PD-L1 inhibitors had regulatory efficacy for metastatic UC patients whose disease progressed following platinum-based chemotherapy [40]. Recently, Bellmunt and his colleagues [7] reported that pembrolizumab (antibody against PD-1) could improve overall survival (by nearly 3 months) and have less therapy-related adverse events than chemotherapy for platinum-resistant advanced UC. Moreover, in a multicenter phase 1 and 2 cohort trial, nivolumab (one PD-1 inhibitor) raised a response in 24.4% of metastatic UC patients who had received previous chemotherapy without regard to TC PD-L1 expression [13, 25, 26, 28]. And their findings also provided that PD-L1 status in immune cells was a promising predictor for selected UC patients treated with atezolizumab or nivolumab. Our results demonstrated that higher PD-L1 expression of TIICs but not TCs with a cut-off value of 5% showed better response to anti-PD-1/PD-L1 immunotherapy. When T-cells were activated by antigen, they produced several cytokines which could increase the expression of PD-L1 in adjacent tumour and immune cells [41]. PD-L1 expression in TIICs as well as TILs was tumour antigen-specific, and their response to the tumour could be an explanation for this. Balar et al. reported a median PFS of 4.1 months in IC2/3 patients which was longer than 2.1 months in IC1 patients and 2.6 months in IC0 patients. However, IC2/3 patients did not benefit from OS with a median of 15.9 months versus 19.1 months in IC0/1 patients [13]. Finally, our meta-analysis convinced us that immune cell PD-L1 status was useful as a prognostic biomarker for PFS but not OS in UC patients receiving anti-PD-1/PD-L1 immunotherapy. Our results supported that PD-L1 expression in immune cells might serve as a promising predictor for immune checkpoint blockade therapies in UC. Furthermore, PD-L1-negative patients also responded to the PD-1/PD-L1 blockade therapy, which highlighted the need for better response biomarkers for immunotherapies [13]. In addition, few studies with short-term follow-up resulted in lack of power for the analysis of positive IC PD-L1 status on survival benefit. Patients should be followed up to assess the response and other long-term survival.

Notably, several limitations still existed in our study. The main limitation of our meta-analysis reflected the drawbacks of the literatures concerning this topic; several available publications were out-of-date or enrolled a relatively small sample size, and only three RCTs were methodologically qualified. The second limitation was the different locations of PD-L1 protein expressed; in most cases, they were only measured in tumour cells. However, there was no study reporting on immune cell PD-L1 status and survival after cystectomy, and only one study evaluated this in UC patients with chemotherapy. We could not pool the conclusion of immune cell PD-L1 status for predicting response and prognosis in patients treated with cystectomy or chemotherapy. The third limitation of our study was few comparative data for survival, leading to the lack of power for the analysis of survival benefit based on the limited studies with small sample sizes and short-term follow-up period. Patients should be followed up to assess the response and other long-term survival. Finally, other limits mainly included the different populations enrolled, the different drugs used, and the varying durations of treatment in the different studies included. Nevertheless, this systematic review offered a comprehensive overview of the predictive and prognostic significance of PD-L1 expression in UC patients after cystectomy, chemotherapy, or anti-PD-1/PD-L1 immunotherapy for data extraction with a robust search strategy. Furthermore, we applied a rigorous inclusion/exclusion criterion to identify studies, different subgroup analyses, full outcomes of interest (ORR, CR, PR, SD, PD, OS, CSS, and DFS), and advanced meta-analysis using HR and corresponding 95% CI for survival. Here, we provided up-to-date information of predictive and prognostic significance of PD-L1 in UC which was worthy for reference to the ongoing clinical trials.

Tumour cell PD-L1 expression showed significant association with advanced UC and could predict survival after cystectomy for organ-confined UC patients. Tumour cell PD-L1 status had no predictive or prognostic benefit from platinum-based chemotherapy. Higher PD-L1 expression of TIICs but not TCs with a cut-off value of 5% predicted better response to anti-PD-1/PD-L1 immunotherapy. TIIC PD-L1 status was useful as a prognostic biomarker for PFS but not OS in UC patients receiving anti-PD-1/PD-L1 immunotherapy. However, further RCTs with longer follow-up and a larger sample size should be conducted to verify whether the tumour immune cell PD-L1 as a biomarker has predictive and prognostic value in advanced UC patients treated with immune checkpoint inhibitors.

## Figures and Tables

**Table 1:** Characteristics of the included studies on PD-L1 status predicting the prognosis in UC patients after cystectomy.

Study	Boorjian et al. (2008) [4]	Wang et al. (2009) [18]	Xylinas et al. (2014) [19]
Country	USA	China	USA
Study interval	1990-1994	2000-2002	1988-2003
Age (years)	69 (37-90)	62 (42-78)	66 (61-72)
Male/female	259/59	40/10	244/58
Management	RC	RC	RC
PD-L1 expression	Tumour cells	Tumour cells	Tumour cells
Detection method	IHC	IHC	IHC
Cut-off value	5%	10%	5%
Follow-up (mons.)	164 (1-210)	28 (6-52)	120 (78-125)
Clinical outcomes	Receipt of BCG, tumour stage, TIL	Tumour grade, tumour stage, recurrent UC	None^∗^
All UC patients			
PD-L1+	12.4%	72.0%	25.2%
PD-L1+/-	39/275	36/14	76/226
OS, HR(95% CI)	1.06(0.71-1.58)	2.24(1.16-4.38)	0.98(0.73-1.31)
*P* value	0.88	0.01	0.79
CSS, HR(95% CI)	0.82(0.42-1.63)	na	0.79(0.53-1.61)
*P* value	0.23	na	0.58
DFS, HR(95% CI)	0.83(0.43-1.58)	na	0.74(0.50-1.11)
*P* value	0.15	na	0.56
Organ-confined disease		na	
PD-L1+	16.2%	na	25.0%
PD-L1+/-	27/140	na	24/72
OS, HR(95% CI)	2.18(1.26-3.77)	na	1.93(1.09-3.43)
*P* value	0.02	na	0.005
CSS, HR(95% CI)	1.59(0.56-4.49)	na	1.21(0.47-3.13)
*P* value	0.68	na	0.38
DFS, HR(95% CI)	1.24(0.44-3.45)	na	1.27(0.50-3.26)
*P* value	0.62	na	0.69

UC: urothelial carcinoma; PD-L1: programmed death-ligand 1; +/-: positive/negative; RC: radical cystectomy; TIL: tumour-inflating lymphocyte; IHC: immunohistochemistry; BCG: Bacillus Calmette-Guerin; OS: overall survival; CSS: cancer-specific survival; DFS: disease-free survival; HR: hazard ratio; CI: confidence interval; na: data not available. ^∗^No association of PD-L1 expression with clinicopathologic features.

**Table 2:** Characteristics of the included studies on predictive and prognostic value of PD-L1 status in UC patients treated with platinum-based chemotherapy.

Study	Baras et al. (2016) [21]	Bellmunt et al. (2015) [3]	Erlmeier et al. (2016) [20]
Country	USA	USA	Germany
UC patients	MIUC	Metastatic UC	Locally advanced
Tumour stage	All stage	pT2-T4	pT3/pT4
Management	TURBT	TURBT, RC	RC
Chemotherapy	Neoadjuvant platinum-based	Adjuvant platinum-based	Adjuvant platinum-based
PD-L1 expression	Tumour cells	Tumour cells/TIICs	Tumour cells
Detection method	IHC	IHC	IHC
Cut-off value	1%, 5%	5%	10%
PD-L1+	20.6%	16.3%	35.5%
PD-L1+/-	7/34	14/86	11/31
ORR (PD-L1+/-)	42.9%/38.2%	na	37.5%/35.7%
OS rate (PD-L1+/-)	na	64.3%/39.5%	54.5%/51.6%

UC: urothelial carcinoma; MIUC: muscle-invasive UC; PD-L1: programmed death-ligand 1; +/-: positive/negative; TURBT: transurethral resection of bladder tumour; RC: radical cystectomy; TIICs: tumour-inflating immune cells; IHC: immunohistochemistry; ORR: objective response rate; OS: overall survival; pT: physical stage; na: data not available.

**Table 3:** Characteristics of the included studies on predictive and prognostic value of tumour-infiltrating immune cell PD-L1 status in UC patients treated with anti-PD-1/PD-L1 therapy.

Study	Balar et al. (2017) [13]	Powles et al. (2014) [24]	Rosenberg et al. (2016) [28]	Petrylak et al. (2018) [27]
Trial name	IMvigor210	PCD4989g	NCT02108652	NCT01375842
Study design	MRCT	MRCT	MRCT	MRCT
Trial phase	Phase 2	Phase 1	Phase 2	Phase 1
Study interval	2014-2015	2011-2013	May 2014-Nov 2014	Mar 2013-Aug 2015
UC patients	Locally advanced and metastatic UC	Metastatic UC	Locally advanced and metastatic UC	Metastatic UC
Age (years)	73 (51-92)	65 (36-86)	66 (32-91)	66 (36-89)
Male/female	96/23	46/19	241/69	72/23
Immunotherapy	Atezolizumab	Atezolizumab	Atezolizumab	Atezolizumab
Target	Anti-PD-L1	Anti-PD-L1	Anti-PD-L1	Anti-PD-L1
Treatment	1200 mg, iv every 3 weeks	15 mg/kg, iv every 3 weeks	1200 mg, iv every 3 weeks	15 mg/kg, iv every 3 weeks
PD-L1 expression	TIICs	Tumour cells/TIICs	TIICs	TIICs
Detection method	IHC	IHC	IHC	IHC
Cut-off value	1%, 5%	1%, 5%	1%, 5%	5%
PD-L1+ (5%/1%)	67.2%/26.9%	81.5%/46.2%	66.8%/32.3%	52.6%
PD‐L1 ≥ 5% vs. PD‐L1 < 5%				
No. of PD-L1+/-	32/87	30/35	100/210	50/44
ORR (PD-L1+/-)	28.1%/20.7%	43.3%/11.4%	26.0%/9.0%	40.0%/11.0%
CR (PD-L1+/-)	12.5%/8.0%	na	11.0%/1.9%	16.0%/2.0%
PR (PD-L1+/-)	15.6%/12.6%	na	15.0%/7.1%	24.0%/9.0%
SD (PD-L1+/-)	na	26.7%/37.1%	16.0%/20.5%	18.0%/21.0%
PD (PD-L1+/-)	na	26.7%/37.1%	44.0%/54.8%	34.0%/55.0%
PD‐L1 ≥ 1% vs. PD‐L1 < 1%				
No. of PD-L1+/-	80/39	53/12	207/103	na
ORR (PD-L1+/-)	23.8%/20.5%	30.2%/8.3%	17.9%/7.8%	na
CR (PD-L1+/-)	10.0%/7.7%	na	6.3%/1.9%	na
PR (PD-L1+/-)	13.8%/12.8%	na	11.6%/5.8%	na
SD (PD-L1+/-)	na	30.2%/41.7%	1.9%/24.3%	na
PD (PD-L1+/-)	na	30.2%/41.7%	51.7%/50.5%	na
PFS(PD-L1+/-) (mon.)	4.1 ± 2.4/2.4 ± 0.6	na	4.0 ± 0.6/2.6 ± 0.3	5.5(2.7-10.8)/1.4(1.3-2.7)
OS rate (PD-L1+/-)	52%/59%	na	48%/30%	42%/10%
Follow-up (mons.)	17.2 (0.2-23.5)	4.2 (1.1-8.5)	11.7 (11.4-12.2)	37.8 (0.7-44.4)


TCs: tumour cells; TIICs: tumour-inflating immune cells; UC: urothelial carcinoma; PD-L1: programmed death-ligand 1; +/-: positive/negative; MRCT: multicenter randomized controlled trial; IHC: immunohistochemistry; ORR: objective response rate; CR: completed response; PR: partial response; SD: stable disease; PD: progressive disease; PFS: progression-free survival; OS: overall survival; na: data not available.

**Table 4:** Characteristics of the included studies on predictive and prognostic value of tumour PD-L1 status in UC patients treated with anti-PD-1/PD-L1 therapy.

Study	Plimack et al. (2017) [23]	Powles et al. (2014) [24]	Sharma et al. (2016) [25]	Sharma et al. (2017) [26]	Patel et al. (2018) [22]
Trial name	KEYNOTE-012	PCD4989g	CheckMate 032	CheckMate 275	NCT01772004
Study design	MRCT	MRCT	MRCT	Nonrandomised	MRCT
Trial phase	Phase 1b	Phase 1	Phase 1/2	Phase 2	Phase 1
Study interval	May 2013-Dec 2013	2011-2013	2014-2015	Mar 2015-Oct 2015	Sept 2014-Mar 2016
UC patients	Locally advanced or metastatic UC	Metastatic UC	Recurrent metastatic UC	Metastatic UC	Locally advanced or metastatic UC
Age (years)	70 (44-85)	65 (36-86)	65.5 (31-85)	66 (38-90)	68 (63-76)
Male/female	23/10	46/19	54/24	211/59	178/71
Immunotherapy	Pembrolizumab	Atezolizumab	Nivolumab	Nivolumab	Avelumab
Target	Anti-PD-1	Anti-PD-L1	Anti-PD-1	Anti-PD-1	Anti-PD-L1
Treatment	10 mg/kg, iv every 2 weeks	15 mg/kg, iv every 3 weeks	3 mg/kg, iv every 2 weeks	3 mg/kg, iv every 2 weeks	10 mg/kg, iv every 2 weeks
PD-L1 expression	Tumour cells	Tumour cells/TIICs	Tumour cells	Tumour cells	Tumour cells
Detection method	IHC	IHC	IHC	IHC	IHC
Cut-off value	1%	1%, 5%	1%, 5%	1%	5%
PD-L1+ (5%/1%)	84.0%	43.1%/10.8%	46.0%/30.6%	37.3%	33.0%
PD‐L1 ≥ 5% vs. PD‐L1 < 5%					
No. of PD-L1+/-		7/58	81/184	na	63/76
ORR (PD-L1+/-)	na	28.6%/25.9%	28.4%/15.8%	na	24%/13%
CR (PD-L1+/-)	na	na	4.9%/1.1%	na	10%/3%
PR (PD-L1+/-)	na	na	23.5%/14.7%	na	14%/11%
SD (PD-L1+/-)	na	14.3%/34.5%	28.4%/20.1%	na	29%/20%
PD (PD-L1+/-)	na	57.1%/29.3%	25.9%/45.1%	na	29%/50%
PD‐L1 ≥ 1% vs. PD‐L1 < 1%					
No. of PD-L1+/-	21/4	28/37	122/143	25/42	na
ORR (PD-L1+/-)	23.8%/0%	28.6%/24.3%	23.8%/16.1%	24.0%/26.2%	na
CR (PD-L1+/-)	na	na	1.6%/0.7%	16.0%/2.4%	na
PR (PD-L1+/-)	na	na	19.7%/15.4%	8.0%/23.8%	na
SD (PD-L1+/-)	na	28.6%/35.1%	28.7%/17.5%	32.0%/26.2%	na
PD (PD-L1+/-)	na	35.7%/29.7%	30.3%/46.9%	32.0%/42.9%	na
PFS(PD-L1+/-) (mon.)	na	na	na	na	11.9(6.1-18)/6.1(5.9-8)
OS rate (PD-L1+/-)	na	na	na	na	59%/51%
Follow-up (mons.)	13 (5-23)	4.2 (1.1-8.5)	15.2 (12.9-16.8)	7.0 (3.0-8.8)	9.9 (4.3-12.1)

TCs: tumour cells; TIICs: tumour-inflating immune cells; UC: urothelial carcinoma; PD-L1: programmed death-ligand 1; +/-: positive/negative; MRCT: multicenter randomized controlled trial; IHC: immunohistochemistry; ORR: objective response rate; CR: completed response; PR: partial response; SD: stable disease; PD: progressive disease; PFS: progression-free survival; OS: overall survival; na: data not available.
